# An Experimental Study: Benefits of Digoxin on Hepatotoxicity Induced by Methotrexate Treatment

**DOI:** 10.1155/2021/6619844

**Published:** 2021-11-10

**Authors:** Banu Taskin, Mümin Alper Erdoğan, Gürkan Yiğittürk, Sibel Alper, Oytun Erbaş

**Affiliations:** ^1^Department of Dermatology, Koc University Hospital, Istanbul, Turkey; ^2^Department of Physiology, Faculty of Medicine, Izmir Katip Celebi University, Izmir, Turkey; ^3^Department of Histology and Embryology, Faculty of Medicine, Mugla University, Muğla, Turkey; ^4^Department of Dermatology, Faculty of Medicine, Koc University, Istanbul, Turkey; ^5^Department of Physiology, Faculty of Medicine, Istanbul Bilim University, Istanbul, Turkey

## Abstract

**Purpose:**

The aim of the study is to examine the possible therapeutic effects of a known cardiac glycoside, digoxin, on a rat model of MTX-induced hepatotoxicity.

**Methods:**

The study was conducted on twenty-four male rats. While eighteen rats received a single dose of 20 mg/kg MTX to obtain an injured liver model, six rats constituted the control group. Also, the eighteen liver toxicity model created rats were equally divided into two groups, one of which received digoxin 0.1 mg/kg/day digoxin (Group 1) and the other group (Group 2) was given saline (% 0.9NaCl) with a dose of 1 ml/kg/day for ten days. Following the trial, the rats were sacrificed to harvest blood and liver tissue samples to determine blood and tissue MDA, serum ALT, plasma TNF-*α*, TGF-*β*, IL-6, IL-1-Beta, and PTX3 levels.

**Results:**

MTX's structural and functional hepatotoxicity was observable and evidenced by relatively worse histopathological scores and increased biochemical marker levels. Digoxin treatment significantly reduced the liver enzyme ALT, plasma TNF-*α*, TGF-*β*, PTX3, and MDA levels and decreased histological changes in the liver tissue with MTX-induced hepatotoxicity in the rat model.

**Conclusion:**

We suggest that digoxin has an anti-inflammatory and antihepatotoxic effect on the MTX-induced liver injury model.

## 1. Introduction

Methotrexate (MTX) is used to treat various skin diseases, inflammatory diseases, and malignancies. In recent years, the use of biological therapies for many cutaneous disorders is trending. However, clinicians continue to use MTX as a significantly less expensive and effective treatment agent [1].

MTX has anti-inflammatory, antiproliferative, and immunosuppressive properties. It is a folic acid analog that binds to dihydrofolate reductase (DHFC) irreversibly and inhibits the formation of reduced folates, which are necessary cofactors for many enzymes. This fact leads to the inhibition of thymidylate synthase activity, which is required for DNA synthesis [2–4]. By the inhibition of DNA synthesis, MTX limits epithelial hyperplasia, consolidates the apoptosis of activated T cells, inhibits the chemotaxis of neutrophils [5], and decreases the synthesis of many proinflammatory cytokines [4].

MTX treatment has significant adverse effects. The most commonly occurring side effect is hepatotoxicity, which is also the most critical one. Chronic MTX use has the potential to induce a variety of hepatic histological changes, such as steatosis, stellate cell hypertrophy, anisonucleosis, and hepatic fibrosis toxicity increases with the total cumulative dose [6]. The mechanisms that cause MTX hepatotoxicity are not precise. Oxidative stress is thought to be responsible for MTX-induced hepatic injury in recent studies [7]. However, the role of interleukin 17A (IL-17A) in liver fibrosis was examined by very few studies [8, 9].

Digoxin can be used to treat atrial fibrillation and heart failure to decrease the ventricular rate. Digoxin increases intracellular calcium in myocardial cells indirectly, by inhibiting the sodium-potassium pump in the cell membrane [10]. Recently, it has been demonstrated that digoxin suppressed murine Th17 cell differentiation without affecting the differentiation of other T cell lineages [11, 12].

The present study is aimed at assessing the antihepatotoxic effect of digoxin on a rat model of MTX-induced hepatotoxicity in rats.

## 2. Methods

### 2.1. The Experiment Protocol

In this study, 24 mature Sprague-Dawley breed male albino rats were used. The weight range of the rats was between 200 and 220 grams. Animals were housed in pairs in steel cages under a temperature-controlled environment (22 ± 2°C) with 12 h light/dark cycles. They were fed ad libitum. The Animal Ethics Committee approved the experimental procedures used in this study. The *Guide for the Care and Use of Laboratory Animals* was followed while conducting the experiments, which was confirmed by the National Institutes of Health (U.S.).

**Figure 1:**
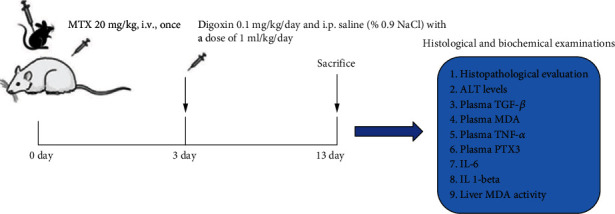
Graphical abstract.

We followed the methods of Taskin et al. [13]. Twenty-four male rats were included in the study. While eighteen rats received a single dose of 20 mg/kg MTX to obtain an injured liver model, six rats constituted the control group. After 3 days of MTX injection, the eighteen rats which are liver toxicity model created were equally divided into two groups, one of which received digoxin 0.1 mg/kg/day (Group1) intraperitoneally (i.p.) and the other group (Group 2) was given i.p. saline (% 0.9NaCl) with a dose of 1 ml/kg/day for ten days (Figure 1). At the end of the study (Day 13), all animals were sacrificed through cervical dislocation under anesthesia (100 mg/kg, Ketasol, Richter Pharma)/xylazine (50 mg/kg, Rompun, Bayer). Blood samples were withdrawn via cardiac puncture and used for biochemical examination. The liver was tissue extracted for histopathological and biochemical examination.

### 2.2. Dose Selection

The dose that we used in the study was chosen regarding the literature and previous animal MTX toxicity models. These doses are necessary for rats to account for their faster drug metabolism, which can easily exceed that of a human by a multiple of ≥10. The MTX dose that we choose for our study can be seen from some previous studies [13–24].

The dosing of these drugs for murine research has been well studied and used between 10 and 1000 *μ*g/kg doses, and in this study, we followed established conventions of this drug dosing. In our study, we used a 0.1 mg/kg (100 *μ*g/kg) dose for digoxin treatment. Regarding the digoxin dose that we chose for our study, you can see the previous studies from the literature [25–29]. There is also a Guidance on Default assumptions used by the EFSA Scientific Panels and Committee and EFSA Units in the absence of actual measured data by the EFSA Scientific Committee [30].

### 2.3. Histopathological Evaluation

Formalin-fixed rectum and colon sections (4 *μ*m) were stained with hematoxylin and eosin. The photographs of all sections were shooted via an Olympus C-5050 digital camera positioned on the Olympus BX51 microscope.

Liver histopathological scoring analysis was based on the Lobenhofer et al. study. The sum of the individual score grades was as follows: 1 (minimal), 2 (mild), 3 (moderate), and 4 (marked) for each of the following parameters from liver sections: hepatocyte necrosis, fibrosis, and cellular infiltration [31].

### 2.4. Biochemical Evaluation

An enzyme-linked immunosorbent assay (ELISA) kit (Biosciences) was used for determining the plasma TNF-*α* level. A ratio of 1 : 2 was used for the dilution of the samples, and TNF-*α* was determined in duplicate following the manufacturer's guide. The detection range for the TNF-*α* assay was <2 pg/ml.

Plasma TGF-*β*, IL-6, and IL-1-Beta were measured using a commercially available enzyme-linked immunosorbent assay (ELISA) kit (Biosciences). TGF-*β* levels were expressed as pg/ml.

A commercially available (ELISA) kit (USCN, Life Science Inc.) was used to detect plasma ALT levels.

Malondialdehyde (MDA) levels as a thiobarbituric acid reactive substance (TBARS) measurement in both tissue and plasma samples were used to determine lipid peroxidation [32]. Trichloroacetic acid and TBARS reagent were added to the tissue samples. Following the combination, the mixture was incubated at 100°C for 60 minutes. After this process, the samples were cooled on ice and centrifuged at 3000 rpm for 20 min. The supernatant absorbance measurement was performed at 535 nm. Tissue MDA levels were calculated from the standard calibration curve using tetra-ethoxy-propane and expressed as nmol/gr protein.

The PTX3 kit (USCN, Life Science Inc., Wuhan, China) was used to measure the plasma pentraxin-3 (PTX3) levels with standard ELISA apparatus at 450 nm. PTX3 levels were obtained under the guidance of the kit's manufacturer.

### 2.5. Statistical Analysis

SPSS version 15.0 for Windows was used for statistical analyses. Student's *t*-test and analysis of variance (ANOVA) were used to compare parametric variables of the groups. The Mann–Whitney *U* test was used for the comparison of nonparametric variables. The results were presented as the mean ± standard error of the mean (SEM). A *p* value < 0.05 was accepted as the significance level, while a *p* value of <0.001 was accepted as a high statistical significance.

## 3. Results

### 3.1. Histological Analysis

The group's histological injury scores are shown in Table 1.

**Figure 2:**
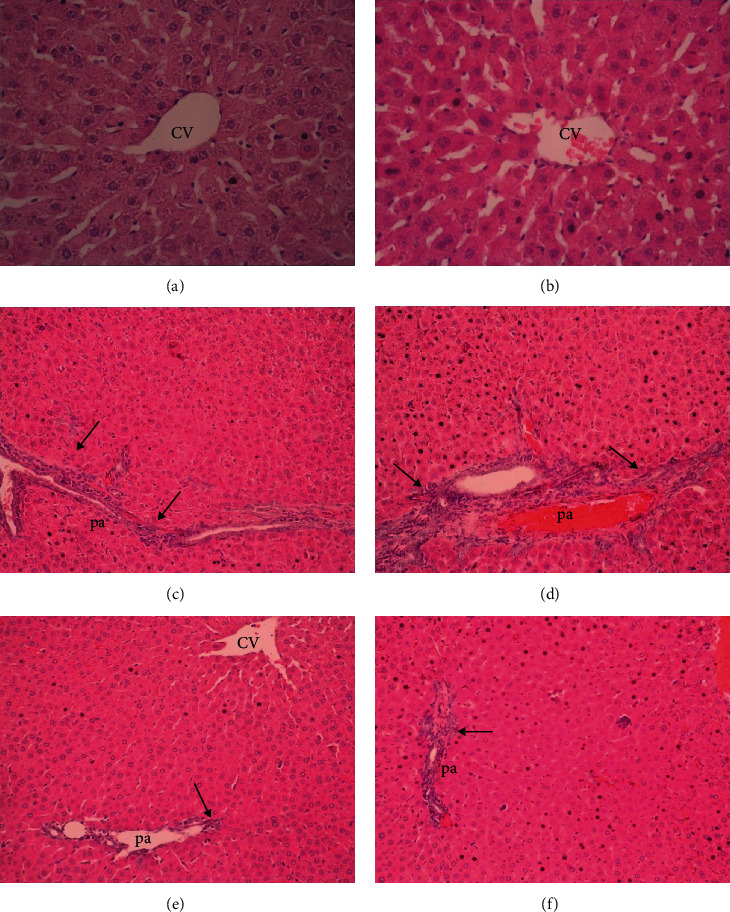
Liver histopathology H&E (×20 magnification): (a, b) normal liver; (c, d) bridging necrosis, fibrosis, and cellular infiltration in the portal area (pa) (arrow), central venous (cv); (e, f) decreased bridging necrosis, fibrosis, and cellular infiltration in the portal area (pa) (arrow).

The normal group's liver sections' histological appearance had healthy liver tissue findings (Figures 2(a) and 2(b)). The histological appearance of the liver sections from the MTX+digoxin group (Figures 2(e) and 2(f)) is significantly better than that of the liver sections from the MTX+saline group (Figures 2(c) and 2(d)).

### 3.2. Biochemical Analysis

The biochemical analysis of the groups is given in Table 2. The MTX+saline group had significantly higher ALT levels, plasma TGF-*β*, plasma MDA, plasma TNF-*α*, plasma PTX3, IL-6, IL-1-Beta, and liver MDA activity compared to the MTX+digoxin group.

## 4. Discussion

Hepatotoxicity can be seen as a side effect of various medications and is also the most severe adverse effect of long-term MTX treatment. The treatment and possible approaches to reduce hepatotoxicity risk are necessary for the improvement of life quality and the success of the treatment [33]. Herein, this experimental study is investigating the digoxin effect on MTX-induced hepatotoxicity in rats.

Various liver histological changes such as hepatocyte necrosis, fibrosis, and increased cellular infiltration were seen in the MTX-treated group, as confirmed with previous studies [33, 34]. We also examined the correlation between histological changes and biochemical tests in addition to creating an injured liver tissue model.

An increase in serum ALT was shown after MTX exposure in prior studies [33, 34]. In accordance with previous studies, we clearly have shown that MTX treatment triggers a path that increases ALT serum levels [34–36]. In our study, we also have shown the increase in systemic inflammatory response indicator TNF-*α* due to MTX administration and show a tendency to decrease and get back to normal ranges by digoxin treatment. MTX administration caused an increase in serum TGF-*β*. This fact highlights the role of the cytokine in MTX-induced hepatotoxicity. In chronic liver diseases, TGF-*β* acts as the central regulator and contributes to disease progression from initial liver injury to fibrosis and carcinoma [37]. Oppositely, digoxin acts as the injury reverser and strains the TGF-*β* response.

We observed that MTX administration increased both the plasma and tissue MDA. Hadi et al. [38] also have shown an increase in serum and tissue MDA levels due to MTX. On the other hand, numerous studies have shown antioxidants' effect on reducing high MDA levels [33]. Our study shows that digoxin administration could also act as an antioxidant and reduce oxidant parameters (tissue and plasma MDA levels).

In our experimental observations, we have seen that MTX exposure led to an increase in plasma PTX3, a glycoprotein that belongs to the PTX family, and has a vital role in the primary inflammatory response [39]. In recent years, PTX3 has been in use as a potential biomarker of hepatic pathologies [40, 41]. We suggest that PTX3 can be a marker of MTX-induced hepatotoxicity as well and can be decreased with digoxin treatment.

The mechanism of MTX-caused hepatotoxicity is not precise. Intracellular accumulation of MTX polyglutamate and consequent folate depletion, generation of oxidative stress, and activation of proinflammatory cytokines, as well as genetic polymorphism, may have a role in this severe result [33, 42]. Specific CD4+ Th cells with proinflammatory properties that differ from Th1 and Th2 cells produce IL-17A. Differentiation of Th17 cells needs IL-1*β*, TGF-*β*, IL-6, and IL23 cytokines, which are induced by activated Kupffer and other cells, promoting the expression of the lineage-specific transcription factor retinoic acid-related orphan nuclear receptor *γ*t (ROR *γ*t: in mice) or ROR c (in humans). ROR *γ*t and ROR c are necessary for the development of Th17 cells [12]. IL-17A plays a critical role in neutrophil recruitment, angiogenesis, inflammation, and autoimmune diseases. Recently, Tan et al. [9] found that IL-17A+ ROR *γ*t+ neutrophils and T cells are applied to the injured liver progressing to chronic, fibrotic hepatitis. IL-17A-dependent hepatic stellate cell activation is critical for liver fibrosis. Several studies have shown that digoxin antagonizes ROR *γ*t receptor activity and suppresses Th17 cell differentiation and IL-17 production [11, 12]. In our study, digoxin treatment significantly decreased MDA levels (plasma and tissue), plasma TGF-*β*, plasma TNF-*α*, plasma PTX3, IL-6, IL-1-Beta, and serum ALT. Digoxin has an anti-inflammatory and antihepatotoxic effect on the MTX-induced liver injury model, and this effect of digoxin can be thought to be due to the suppression of Th17 cell differentiation and IL-17 production. However, no molecular data from studies prove this claim—also, Ouyang and Mahal [43] showed the beneficial effect of digoxin on alcoholic and nonalcoholic steatohepatitis generated by a mouse model given intragastric alcohol. Ouyang et al. [28] also suggested that digoxin binds pyruvate kinase M2 (PKM2), limiting its availability to regulate the transcription of proinflammatory genes. PKM2 is a novel target for the manipulation of sterile inflammation in the liver. Digoxin can protect the liver from a wide range of injuries due to binding PKM2. A significant decrease in IL-6 and IL-1-Beta levels also showed that digoxin may perform its anti-inflammatory effects through inhibition of these proinflammatory cytokines. In the study of Leite et al. [44], their findings were supporting our results in liver tissue. Cardiac glycosides have been found to reduce fluid extravasation (edema formation), leukocyte infiltration, and levels of the cytokines IL-1*β* and tumor necrosis factor a (TNF-*α*), but not leukocyte viability or function (phagocytosis) [44]. Taken together, it can be said that cardiac glycosides suppress acute inflammation in vivo. As a result of these findings, these digoxin mechanisms can initiate new studies regarding its usage for treating liver toxicity and inflammatory diseases.

## 5. Conclusion

As all the histological and biochemical results in our study clearly showed, we evaluate that digoxin treatment has antihepatotoxic effects on MTX-induced hepatotoxicity in a rat model.

## Figures and Tables

**Table 1:** Histological analysis of the results.

	Normal	MTX+saline	MTX+digoxin
Hepatocyte necrosis	0.12 ± 0.1	2.1 ± 0.22^∗^	1.25 ± 0.16^#^
Fibrosis	0.25 ± 0.16	1.75 ± 0.3^∗∗^	0.75 ± 0.25^#^
Cellular infiltration	0.12 ± 0.1	1.25 ± 0.16^∗∗^	0.6 ± 0.18^#^

^∗^ *p* < 0.0001, MTX+saline group compared with normal group. ^∗∗^*p* < 0.01, MTX+saline group compared with normal group. ^#^*p* < 0.05, MTX+digoxin group compared with MTX+saline group.

**Table 2:** Biochemical analysis of the results.

	Normal	MTX+saline	MTX+digoxin
Plasma TGF beta (pg/ml)	6.2 ± 0.5	40.4 ± 5.9^∗^	15.3 ± 1.9^#^
Plasma MDA (nM)	47.3 ± 4.8	208.3 ± 31.4^∗^	87.2 ± 8.8^#^
Plasma TNF-alpha (pg/ml)	19.5 ± 1.8	76.9 ± 3.8^∗^	28.2 ± 4.5^#^
Plasma pentraxin-3 (ng/ml)	1.3 ± 0.1	3.7 ± 0.5^∗^	2.1 ± 0.4^#^
ALT (IU/l)	25.8 ± 2.9	88.3 ± 6.7^∗^	35.1 ± 5.07^#^
Liver MDA (nmol/g tissue)	27.9 ± 3.7	80.9 ± 5.8^∗^	42.35 ± 5.3^#^
IL-6 (pg/ml)	7.1 ± 2.5	21246.1 ± 867.7^∗^	10762.5 ± 492.3^#^
IL-1-Beta (pg/ml)	3.9 ± 0.9	2534.1 ± 148.1^∗^	691.6 ± 48.2^#^

^∗^ *p* < 0.0001, MTX+saline group compared with normal group. ^#^*p* < 0.0001, MTX+digoxin group compared with MTX+saline group.

## Data Availability

The datasets used and analyzed during the current study are available.
